# Evidence of long‐lasting anti‐CD19 activity of engrafted CD19 chimeric antigen receptor–modified T cells in a phase I study targeting pediatrics with acute lymphoblastic leukemia

**DOI:** 10.1002/hon.2672

**Published:** 2019-09-15

**Authors:** Futian Ma, Jin‐Yuan Ho, Huan Du, Fan Xuan, Xiaoli Wu, Qinglong Wang, Lin Wang, Ying Liu, Min Ba, Yizhuo Wang, Jianmin Luo, Jianqiang Li

**Affiliations:** ^1^ Department of Pediatrics Hematology‐Oncology The Second Hospital of Hebei Medical University Shijiazhuang China; ^2^ CAR‐T Research Center Hebei Senlang Biotechnology Co., Ltd. Shijiazhuang China; ^3^ Department of Hematology The Second Hospital of Hebei Medical University Shijiazhuang China

**Keywords:** B‐ALL, CAR‐T, CD19, chimeric antigen receptor

## Abstract

Ninety percent of relapse/refractory B‐cell acute lymphatic leukemia (R/R B‐ALL) patients can achieve complete remission (CR) after CD19‐targeting chimeric antigen receptor T (CAR‐T) cell therapy. However, around 50% of them relapse in 1 year. Persistent CAR‐T cell engraftment is considered as the key to remain durable remission. Here, we initiated a phase I study to treat 10 pediatric B‐ALL patients using a CD19‐targeted second generation CAR with a 4‐1BB intracellular costimulatory domain. All patients received a standard fludarabine and cyclophosphamide (FC) preconditioning regiment, followed by a CAR‐T infusion with a median number of 0.5 (0.3‐1.58) × 10^6^ CAR+ T cells/kg. The pretreatment tumor burdens were high with a median bone marrow (BM) blasts percentage of 59.2% (7.31%‐86.2%), excluding one patient only with brain infiltration of leukemia cells (0% BM blasts). The initial CR rate was 80% (n = 8/10). Four patients (40%) experienced serious (grade > 2) cytokine release syndrome (CRS) and three patients (30%) with obvious neurotoxicity. Monthly assessments of CD19+ minimal residual disease (MRD) and CAR‐T engraftment demonstrated the anti‐CD19 activity of long‐term engrafted CAR‐T cell clones in one patient for more than 2 years.

## 1 INTRODUCTION

Since the first report of genetically engineered T cell with chimeric antigen receptor (CAR‐T), CAR‐T has become the hope for relapsed/refractory patients, especially for those with hematologic malignancy.^1^ To date, more than 130 ongoing registered clinical trials are recruiting patients to investigate the safety and efficacy of variety design of CD19 CAR‐T cells (http://clinicaltrial.gov). Several groups have reported extended regressions of B‐cell malignancies in patients receiving infusions of anti‐CD19 CAR‐T cells at different ages.^2^, ^3^, ^4^, ^5^

Recent studies indicated high initial remission rates of B‐cell acute lymphoblastic leukemias (B‐ALL) in adults with response rate ranging from 83% to 93%.^5^, ^6^, ^7^ In contrast, children's response rate was lower than that of adults, expanding from 68% to 90%.^1^, ^8^, ^9^ Although adults with B‐ALL display 10% higher relapse rates compare with pediatric patients and experience long‐term event‐free survival of less than 50%, it is reported that approximately 25% of pediatric patients have a relapse with CD19 negative or CD19 low and resulted in a considerable number of all childhood cancer deaths.^10^, ^11^ Thus, developing efficient and molecular‐targeted approaches to prolong the life‐span of B‐ALL children has become a vital priority in recent years. CAR‐T cells targeting CD19 has successfully shown tremendous potential for B‐cell lineage malignancies.^2^, ^4^, ^5^, ^12^ Here, we report the safety, efficacy, and correlative studies of CD19 CAR‐T cells therapy in 10 pediatric patients in China.

## 2 RESULTS

### 2.1 Design of CD19 CAR‐T cells and patient enrollment

**Figure 1:**
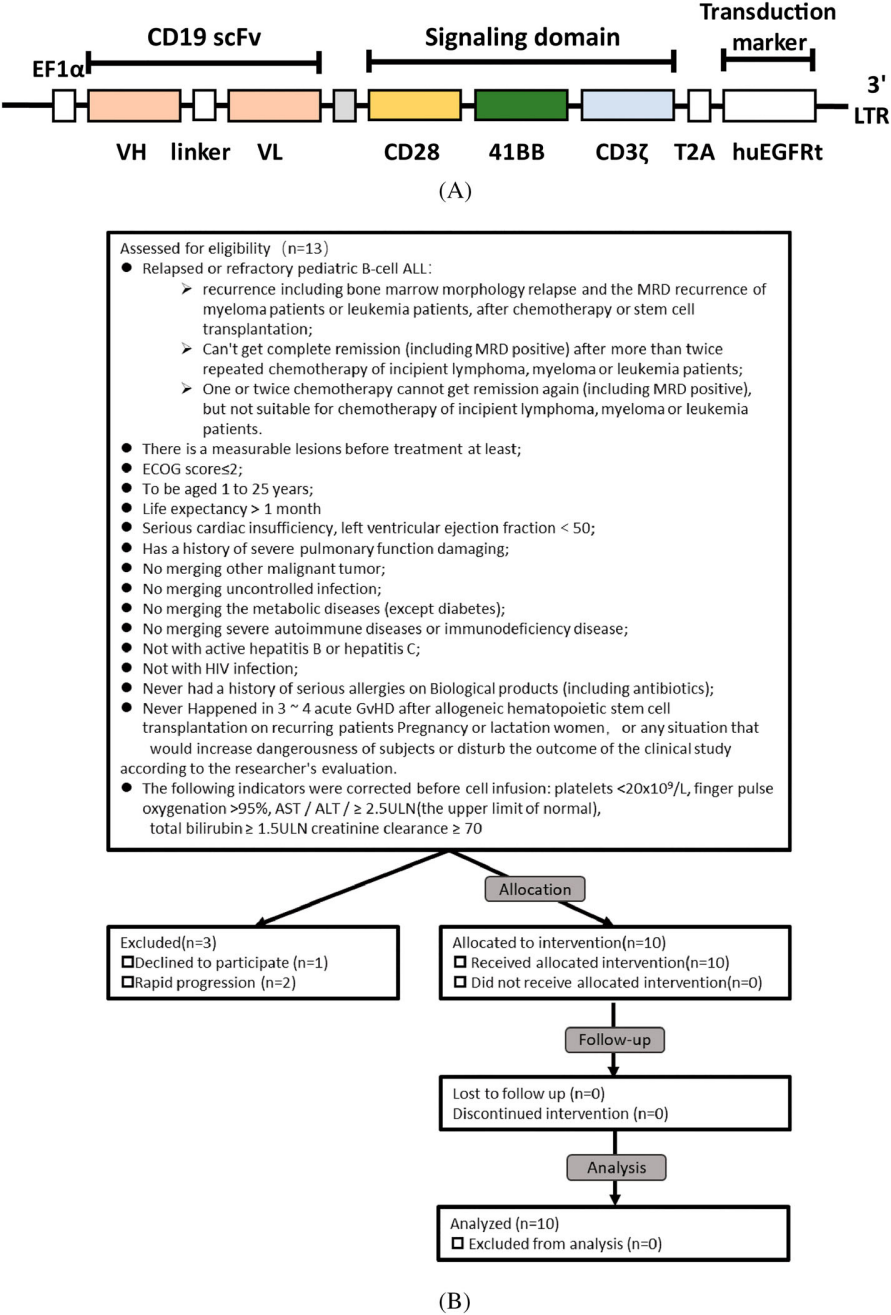
The structure feature of CAR construct (A) including VH, heavy chain variable domain; VL, light chain variable domain, hinge, transmembrane domain, 4‐1BB and CD3 ζ. (B) The screen, enrollment criteria, and the treatment

The sequence of CD19 chimeric antigen receptor is composed by CD19 scFv, CD28‐hing, 4‐1BB costimulatory region, and CD3ζ activation domains (Figure 1A). T cells were activated by CD3/CD28 microbead 2 days before CD19‐CAR lentivirus infection. After transduction, the T cells were expanded for another 10 days before formulation and infusion. The detail of manufacturing of Senl‐B19 was outlined in Figure S1.

### 2.2 Demographics and baseline characteristics

Patients with relapsed or refractory B‐ALL in Hebei Second Provincial People Hospital were recruited in the phase I/II clinical trial of CD19 CAR‐T cell treatment (NCT02963038). The enrollment procedure is outlined in Figure 1B. Patients' characteristics are summarized in Table 1. The median age was 6.5 (range 3‐13 y); none of them has prior hematopoietic stem cell transplant. All patients have experienced at least three courses of chemotherapy prior CAR‐T infusion (median 4, range 3‐10 times). Mean bone marrow (BM) blast was 40.8% (range 0% to 86.2%) at the first day (day −5) of standard preconditioning regiment (fludarabine 25 mg/m^2^; cyclophosphamide 900 mg/m^2^). All patients were carefully observed in the hospital for the first month to monitor the adverse effect and efficacy. Complete blood counts and cytokine concentration were examined at different time course, including day −1 to 0, day 1, day 4 or 5, day 7, day 10 or 11, day 14, day 21, and day 28. Afterwards, levels of CAR‐T cells and residual leukemia cells in BM were analyzed monthly.

**Table 1:** Patient characteristics

Patient No.	Sex	Age at Diagnosis	Weight, kg	Diagnosis/Stage	Disease Status	ECOG PS	Prior Treatment	Philadelphia Chromosome
P03	F	13	40	B‐ALL/IV	Relapsed	1	VDLP, CAM, HDMTX	Ph‐
P04	F	6	26	B‐ALL/IV	Relapsed	2	VDLP, CAM	Ph‐
P09	F	6	20.5	B‐ALL/IV	MRD+	1	VDLP, CAM	Ph‐
P19	M	3	17	B‐ALL/IV	Primary refractory	2	VDLP, VDLP, CAM, VMLP	ND
P21	F	5	22	B‐ALL/IV	Relapsed	2	VDLP, VD, MTX + ARAC + DX, Radiotherapy	Ph‐
P26	M	10	43	B‐ALL/IV	Relapsed	2	VDLP, CAMX*2, HDMTX‐CF*2, HDMTX‐CF + VD, VDLD*2, CAM, VD	Ph‐
P34	M	6	34	B‐ALL/IV	Relapsed	2	VDLP, VDLD + 6MP, COAD, VMLD,	Ph‐
P44	F	11	37	B‐ALL/IV	Relapsed	2	VDLP, VDLD,	Ph‐
P57	F	10	54	B‐ALL/IV	Relapsed	2	VDLP, COAD, MTX + ARAC + DX, MTX, radiation	Ph‐
P68	M	6	21	B‐ALL/IV	Relapsed	2	VDLP, VDLD	Ph‐

Abbreviations: ECOG PS, Eastern Cooperative Oncology Group performance status; MRD+, minimal residual disease positive.

### 2.3 Safety and efficacy

**Figure 2:**
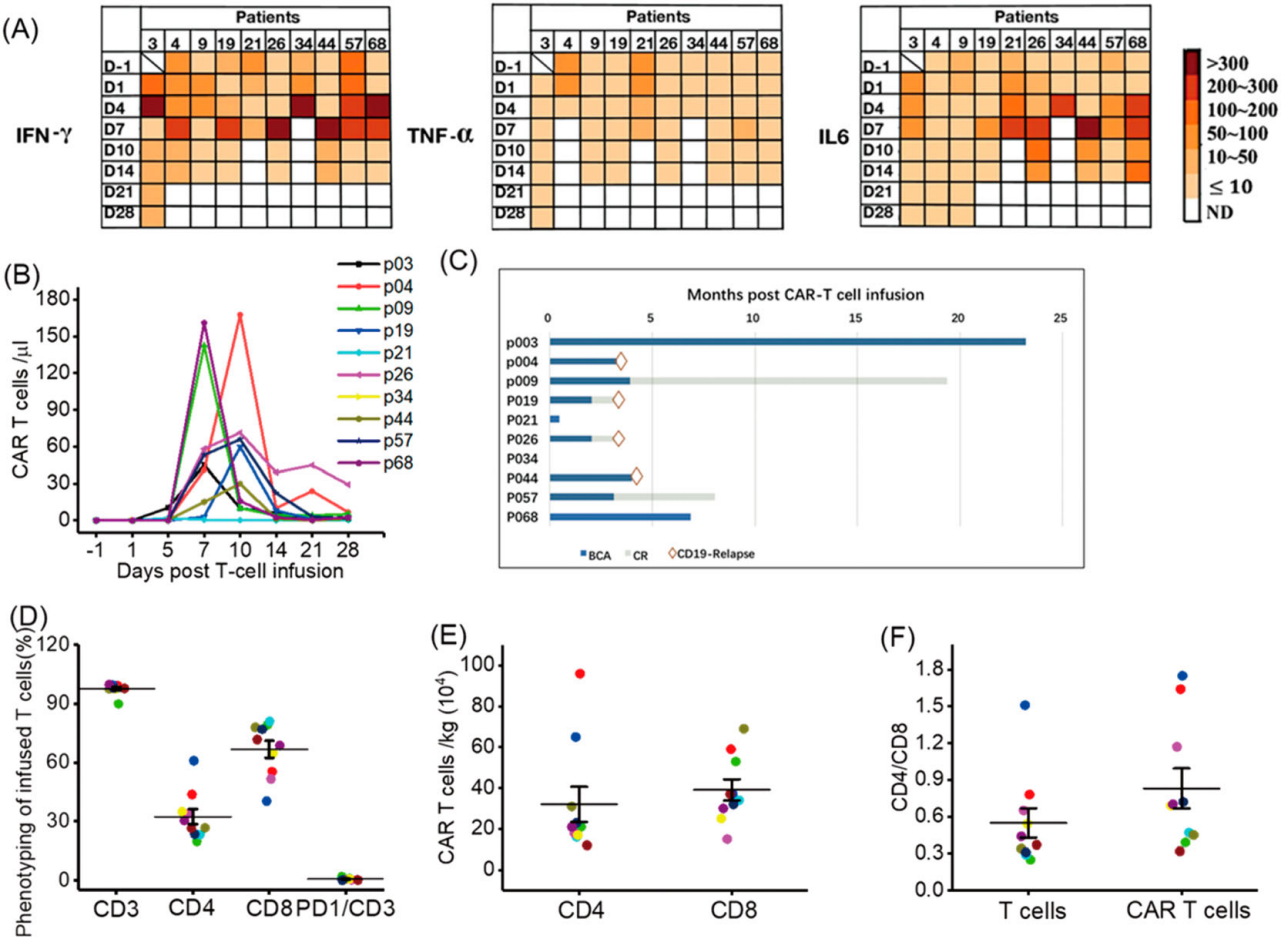
Patient responses after treatment. The dynamic of interferon γ, tumor necrosis factor α, IL‐6 profile (A), and CAR‐T cell expansion (B) within first month. Clinical response of patients after treatment, BCA, B‐cell aplasia; CR, complete remission; red square: CD19‐negative relapse (C). The analysis of infused T cells, T cell phenotyping (D), absolute number of infused cells (E), and the CD4:CD8 ratio of infused total T and CAR‐T cell (F)

The median percentage of BM blasts (prior treatment) was 59.2% (7.31%‐86.2%), excluding one who only with brain infiltration of leukemia cells (0% BM blasts). Patients were infused with various doses of CD19 CAR‐T cells as shown in Table 2. The average dose of treatment was 0.71 × 10^6^ cells/kg (range 0.3‐1.58 × 10^6^ cells/kg), and the average transfection rate was 32.0% ± 24.7%. Most patients received one infusion, except patient 21 infused twice. Cytokine release syndrome (CRS) is the major risk for patients receiving CAR‐T cell therapy especially in hematologic malignancy.^13^ In this report, six patients encountered grade 1/2 CRS, three patients with grade 3 CRS, and one with grade 4 (Table 2). In addition, central nervous system (CNS) neurotoxicity also occurred in three patients (pts 4, 34, and 68). Toxicities were managed by supportive care ± tocilizumab and/or dexamethasone depending on physicians' instruction. Thirteen cytokines were examined, and six of them were with significantly elevated concentration along with CAR‐T cell expansion. The dynamics of cytokine release (TNF‐α, INF‐γ, and IL‐6) were summarized in Figure 2A. The peak of CAR‐T cell expansion was around day 7 to day 10 and rapidly decreased within 5 to 10 days (Figure 2B). Some patients revealed different adverse effects, which were summarized in Table S1.

**Table 2:** Patients' response and toxicity

Patient No.	D‐5 BM Blast (%Leukocytes)	D‐1 PB Blast (%Leukocytes)	Dose of CAR‐T Cells/kg (10^6^)	Dex	Toc	AUC‐60 Days (10^5^)	*C*_max_ Copies/μg DNA (10^5^)	CRS Grade	CNS Grade	Response at First Month	Disease Status at Data Lock	OS Days	PFS Days	BCA Days
P03	7.31	0.34	1.58	N	Y	16.8	2.39	1	0	MRD‐CRi	CRi	734	734	734
P04	77.2	0.80	0.73	Y	Y	2.75	0.23	1	3	MRD‐CR	Relapsed	210	99	99
P09	8.70	0	1.00	N	N	7.57	2.10	1	0	MRD‐CR	CR	620	620	118
P19	59.2	0	0.50	Y	N	4.70	0.56	3	2	MRD‐CR	Relapsed	149	95	61
P21	75.0	63.0	0.30	N	N	1.97	0.08	1	0	PR	Progressed	69	0	14
P26	13.0	0.63	0.50	Y	Y	2.92	0.45	2	2	MRD‐CR	Relapsed	228	96	61
P34	86.2	0.01	1.00	Y	Y	NA	NA	5	3	Death (day 6)	Death	5	0	0
P44	70.0	0.26	0.50	Y	Y	13.3	2.19	3	2	MRD‐CRi	Relapsed	310	120	120
P57	0	0.07	0.50	N	N	8.83	1.61	1	0	MRD‐CR	CR	280	280	94
P68	11.0	4.00	0.50	Y	Y	3.02	0.72	3	4	MRD‐CRi	CRi	244	244	0

Abbreviations: AUC, area under the curve; BM, bone marrow; BCA, B‐cell aplasia; CNS, central nervous system; CR, complete remission; CRi, CR incomplete count recovery; CRS, cytokine release syndrome; Dex, dexamethasone; MRD‐, minimal residual disease negative; OS, overall survive; PB, peripheral blood; PFS, progression‐free survival; PR, partial response; Toc, tocilizumab.

Among the 10 patients, eight of them reached minimal residual disease (MRD) negative after first month (Table 2), except patient 34 and patient 21. Patient 34 received 1 × 10^6^ CAR‐T cells/kg, but he was dead because of rapid disease progression within 5 days. Patient 21 infused CAR‐T cells twice, but the blast was not controllable, diseased 2 months after first infusion. To date, the status of patients was summarized in Figure 2C.

The median overall survival months and event‐free survival months for eight CR patients were 10.3 and 4 months, respectively (Figure S2A,B). The median observation period was 261 days (149‐734). Four patients relapsed, all of them were associated with the loss of CD19 cell‐surface expression, including one lineage switch from ALL to myeloid leukemia. Tumor burdens prior treatment were highly correlated with relapse events. The patient with only brain infiltrating blasts achieved CR and remains for 280 days. The other three patients remaining in remission state have been following up for 244, 620, and 734 days, respectively.

The composition of infused CAR‐T cells was further analyzed (Table S2). The average of CD3+, CD4+, and CD8+ T cells was 97.7%, 32.4%, and 66.8%, respectively (Figure 2D). The expression of PD1 was around 0.24% to 2% (not available for pts 3, 9, and 57), which suggested the activity of infused T cells. Further analysis of the absolute number of CAR‐T cells and CD4/CD8 ratio (Figure 2E,F) revealed that the T cell population of most patients were CD8 dominant.

### 2.4 Overall efficacy and CRS response

**Figure 3:**
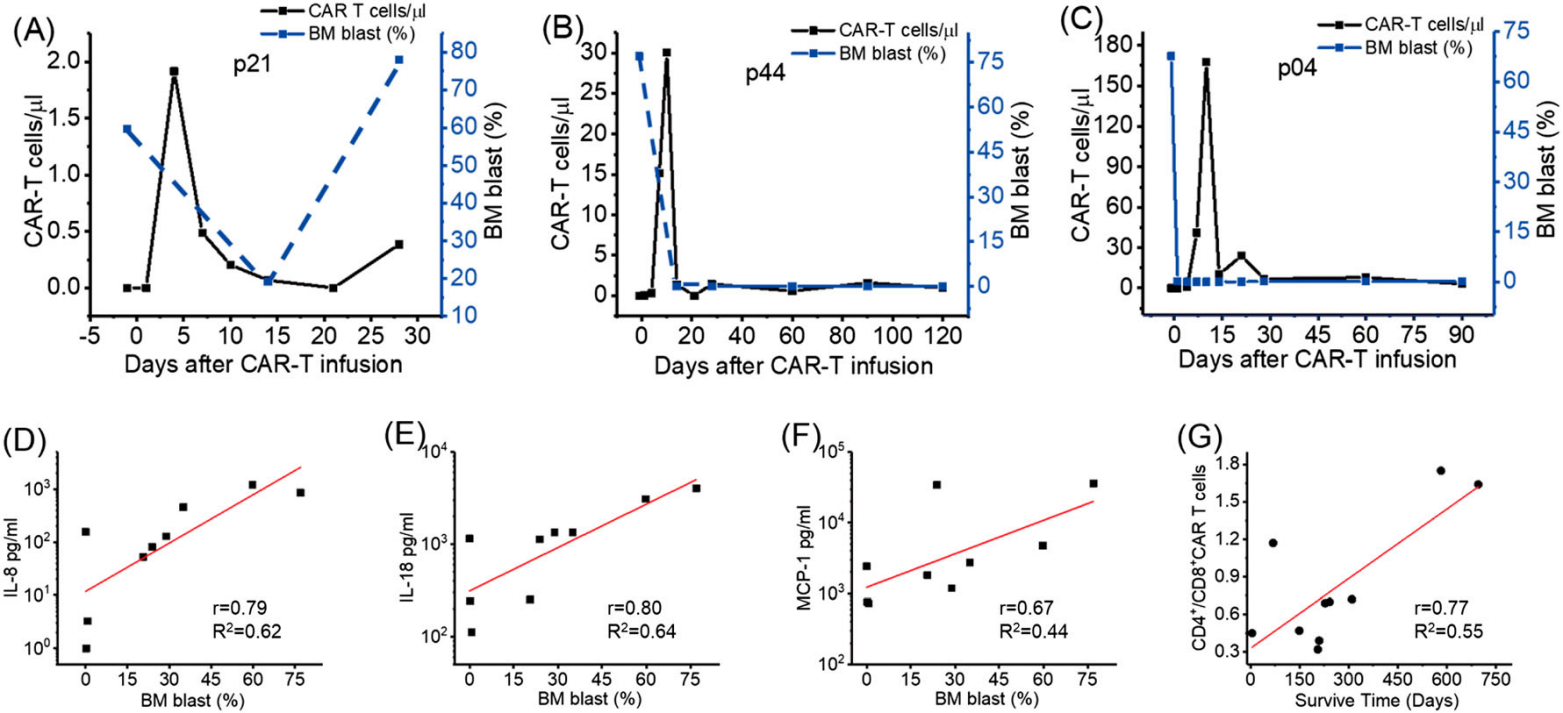
The dynamics of CAR‐T cells number, and the BM blast of three patients with more than 70% blast before infusion, patient 21st (A), patient 44th (B), and patient 4th (C). The correlation of cytokine release and the percentage of bone marrow blast, IL‐8 (D), IL‐18 (E), MCP‐1 (F). The correlation between CD4/CD8 CAR‐T ratio and the length of survival time (G)

Three patients (pts) with more than 70% blast in BM receiving 0.3 (pt 21), 0.5 (pt 44), and 0.73 (pt 4) × 10^6^ CAR‐T cells/kg were further analyzed (Figure 3A‐C). Despite they started with more than 70% blast, two of them, pts 4 and 44, reached minimal residue disease complete remission and CRi (i: incomplete), respectively. This result suggested a correlation between initial response and the dose of infusion.

CRS is suggested to be correlated with the blast amount. However, the dosages of infusion were sometimes adjusted to reduce the chance of severe CRS; thus, we did not observe a strong correlation between BM blast and CRS degree (Figure S3). The peak concentration of IFN‐γ and IL‐6, but not IL‐8 or IL‐18, was slightly correlated with the degree of CRS (Figure S4). In addition, we found that the peak of IL‐8, IL‐18, and monocyte chemoattractant protein–1 (MCP‐1) production was correlated to the blast percentage of the BM (Figure 3D‐F), but not to peripheral blast amount (Figure S5). The peak of other cytokines, including IL‐1β, IFN‐γ, and IL‐6, was not correlated to the blast percentage of neither in BM nor in peripheral blood (PB) (Figure S6). In short, IFN‐γ and IL‐6 concentrations were correlated with the severity of CRS, but there was no clue to know who would develop strong cytokine release except regularly monitored.

### 2.5 Relapse and long‐term survival

In this report, four patients were relapsed around 3 to 4 months after infusion. According to their last results of flow cytometry analysis and qPCR quantitation before relapse, the detection of CAR DNA suggested the persistence of CD19 CAR‐T cells against CD19+ leukemia. The blast phenotypes of patients 4 and 44 were CD19‐/CD22‐; for patient 19, it was CD19‐/CD22+; and for patient 26, it was a myeloid lineage relapse.

On the other hand, we analyze potential factors that may correlate to long‐term survival from many aspects. We found two patients with long‐term survival were happened to be high CD4 ratio (Figure 2E,F, red and blue dots.) We examined the correlation between overall survival time and the ratio of CD4+CAR‐T/CD8+CAR‐T (Figure 3G).

**Figure 4:**
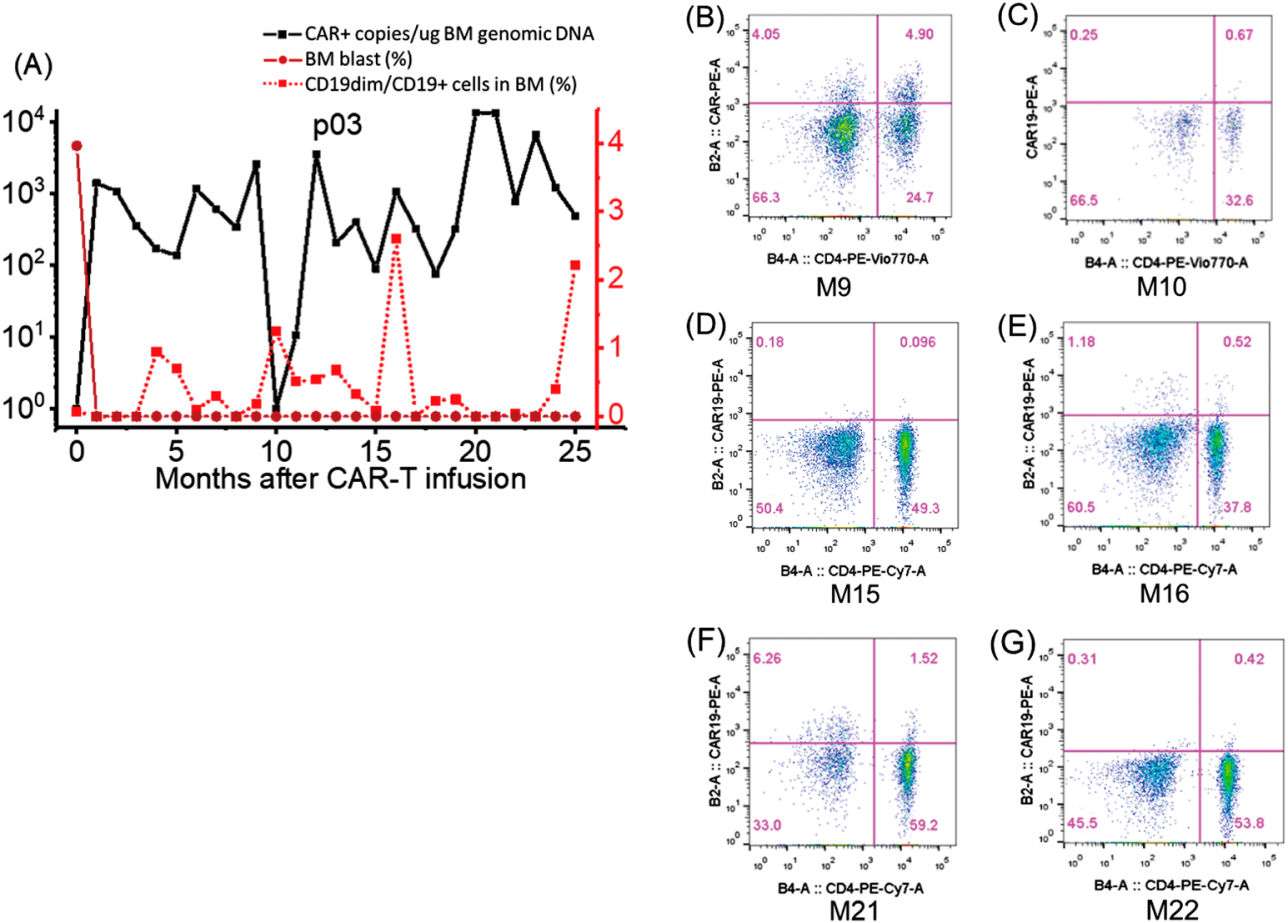
The kinetics of CAR copy number, percentage of BM blast, and the percentage of CD19 positive cells in BM of patient 3 (A). Flow cytometry results of CAR19 in BM samples after 9, 10, 15, 16, 21, and 22 months after treatment

Besides, monthly follow‐up of these two patients revealed several rounds of CAR‐T cell expansion (Figures 4 and S7). As shown in Figure 4A, our first patient who has the longest remission time (734 d), the emergence of CD19+ cells was detected for several times. In addition, we observed a tidal pattern, which showed the increase of CD19+ cells in 1 month would result in the increase of CAR‐T copy number in the next month. Although we still observed the increase of both CAR‐T copy number and CD19+ cells, we considered that may due to timing of examination instead of technical error. The results of flow cytometry further corroborated the increase of CAR‐T copy number; months 9th, 16th, and 21st with higher copy numbers of CAR DNA were also with significant amount of CAR‐T cells (Figure 4B,E,F). The adjacent months, 10th, 15th, and 22nd, showed lower CAR‐T cells (Figure 4C,D,G). Notably, without any further treatment, anti‐CD19 activity was demonstrated obviously by the clearance of CD19+ cells and increase level of CAR‐T cell engraftment even more than 20 month after infusion. An early report revealed an integration‐related mutation on a methylcytosine dioxygenase TET2 gene^14^ resulted in a dominant clone expansion of the specific mutated CAR‐T cell. On the basis of that, we were sequencing the samples of the first patient for T cell population, to understand the mechanism of long‐term persistency.

## 3 DISCUSSION

In this study, despite the high rate of MRD‐negative CR/CRi (8/10, 80%) demonstrates that infusion of our CD19 CAR‐T product is an effective salvage therapy for pediatric and young adults with relapsed/refractory B‐ALL, there was a significant risk of relapse (4/8, 50%) during 3 to 6 months after CAR‐T infusion. CD19‐negative relapses represent a novel mechanism of tumor escape after CD19 CAR‐T treatment while the incidence rate was variable in different studies. In a study run at the Children's Hospital of Philadelphia and at the University of Pennsylvania, 88% (54/59) pediatric patients with r/r B‐ALL achieved MRD negativity after treatment with 2nd generation, 4‐1BB costimulated CAR‐T cells. After 1 year, 20 patients (37%) relapsed and 13 of them (65%) with CD19‐negative disease.^1^ More recently, the Seattle group reported that 10 of 20 relapsed pediatric and young adults after CAR‐T treatment were CD19‐negative disease.^15^ In our study, all four relapsed patients (100%) were CD19 negative including one with myeloid lineage switch. Because of the small size of this study, no clear clues for the high incidence rate of CD19‐negative relapse can be concluded.

Long‐term functional persistence of CAR‐T cells reflected by the duration of B‐cell aplasia (BCA) might increase the chances of antigen‐loss relapse. The Seattle group has observed in six relapsed patients who lost BCA longer than 6 months,^15^ four of them (67%) were CD19 loss that suggests a strong correlation between the incidence of CD19‐negative relapse and longer BCA duration. However, there were still 25% relapsed patients (2/8) who lost BCA in 2 months after CAR‐T therapy were CD19 negative. Therefore, other tumor escape mechanisms besides sustained functional persistence of CAR‐T cells cannot be excluded. The absence of subsequent allo‐SCT may also increase the chances of antigen‐loss relapse. In our study, no CR patients received further therapy including allo‐HCT that might potentially lead to a high rate of CD19‐negative relapse.

To be noted, pt 3 is still under CR with BCA for more than 2 years. The persistence of CAR‐T cells and the retainment of antileukemia functionality were detected as long as 2 years after treatment (Figure 4). Despite the long duration of BCA, we have not observed obvious serious adverse effects correlated with BCA with a closely following up during the past 2 years. During the time, and long‐term persistence of functional CAR‐T cells were detected as long as 2 years after treatment, which is consistent with previous speculate. She only encountered one pneumonia and stayed in the hospital for 10 days. There were no other complications within all these days. On contrary, the other three sustained remission patients lost BCA at 2 to 4 months after CAR‐T infusion (Table 2). Our data have not shown a clear correlation of sustained remission status with the duration of BCA.

Immunologists have been looking for methods to prolong the persistence of CAR‐T cells, including CAR‐T derived from central memory T cells,^16^ confined subset of CD4+ and CD8+ T cells,^17^ or CD4+ T cells alone.^18^ Lately, Wang et al highlight the pivotal role of CD4+ T cells in xenograft models^19^ and consolidated their scheme with the results from their clinical trials. Interestingly, two patients with high CD4/CD8 CAR‐T ratio, in this study, were continuously in CR and BCA, which was in consent with Wang's observation.^19^ This observation suggests that high CD4 ratio may benefit long‐term survival.

To sum up, our study validates the safety and efficacy of CAR‐T cell therapy targeting CD19 in 10 pediatric patients, which encourage us to explore more patients with relapse/refractory B‐ALL in the future. Importantly, antileukemia activity lasting for more than 2 years demonstrates the maintenance of durable remission could be achieved by only single shot of CAR‐T therapy.

## 4 MATERIAL AND METHODS

### 4.1 Generation of the lentiviral construct and package of lentivirus

The CD19‐CAR is composed by anti‐CD19 scFv (FMC63), CD28 transmembrane domain, 4‐1BB, CD3zeta, T2A autocleavage sequences, and endodomain‐deleted EGFR (tEGFR). Three lentivirus package systems were used: psPAX2, pMD2.G, and pLenti‐EF1‐CAR19 were cotransfected with jetPRIME (Polyplus Transfection) in 293FT cells. The medium with lentivirus was collected after 48 hours incubation and then centrifuged to remove cell debris. After 2 hours of 20 000*g* centrifugation, virus pellet was resuspended in TexMACS (Miltenyi Biotec), aliquoted, and stored in −80°C until further use.

### 4.2 Generation of CD19 CAR‐T cells

PBMC from the PB was obtained by Ficoll density centrifugation. T cells were selected by CliniMACS CD3 (Miltenyi Biotec) and then activated by CD3/CD28 microbeads (Gibco) with IL‐2 (200 IU/mL, Miltenyi Biotec) in TexMACS (Miltenyi Biotec). T cells were transduced with CD19‐CAR lentivirus in 24‐well plates. Two days later, the percentage of CAR positive T cells was determined by flow cytometry. And then the CAR‐T cells were transferred to a culture bag on the 6th day and expanded in it for another 10 to 12 days before infusion.

### 4.3 Clinical study

From July 2016 to December 2017, 10 pediatric patients with CD19+ r/r B‐ALL were treated. We used a 2nd generation CAR including a 4‐1BB intracellular domain and a truncated EGFR sequence that can be used to identify and select CAR+ cells. The percentages of MRD were determined by flow cytometry. The adverse effects during and after infusion were assessed according to the NIH Common Terminology Criteria for Adverse Events, version 4 (http://ctep.cancer.gov). Early response was determined according to the National Cancer Institute's International Working Group criteria.

### 4.4 Immunophenotyping

Tumor cells and T cells were phenotyping with CD3‐APC (OKT3, UCH1), CD19‐APC CY7 (SJ26C1), CD4‐PE Cy7 (RPA‐T4, A161A1), CD8‐pacific blue (RPA‐T8, SK1), CD10‐APC (HI10a), CD22‐PE (HIB22), CD38‐PECy7 (HTT2), CD56‐PE CY7 (MEM‐188), CD62L‐BV510 (DREG‐56), PDL1‐PE (29E.2A3), PD1‐FITC (EH12.2H7), streptavidin‐APC‐Cy7, and 7AAD, which were purchased from BioLegend. CD20‐FITC (2H7), CD45RA‐FITC (HI100), and CD62L‐PE (DREG‐56) were purchased from BD. CD137‐PE, CD34‐APC, and antibiotin‐PE were purchased from MACS. The percentage of CAR‐CD19 was determined by biotinylated Erbitux. All cells were analyzed MACSquant with a filter set for eight fluorescence signals and analyzed with FlowJo software (Tree Star).

### 4.5 Quantitation of CD19.CAR transgene

Expression of CD19‐CAR was quantitated with biotinylated Erbitux, which was further labeled with streptavidin‐PE. After CD19 CAR‐T cell infusion, the genomic DNA was extracted from the PB with QIAamp DNA Blood Mini Kit (Qiagen), according to the manufacturer's instruction. The standard curve was established by serial dilution of the plasmid encoding the transgene. The target gene was amplified with WPRE specific primers and probe and normalized with CDKN1a. The amplification was performed by CFX Connect (Bio‐Rad).

### 4.6 Cytokine measurement

The cytokine concentrations, including IL‐1β, IFN‐α2, IFN‐γ, TNF‐α, MCP‐1, IL‐6, IL‐8, IL‐10, IL‐12p70, IL‐17A, IL‐18, IL‐23, and IL33, were determined by BioLegend LEGENDplex human inflammation panel (13‐plex, cat no.740118), according to manufacturer's instruction. In short, 0.025 mL of serum was mixed with 0.025 mL of microbeads and another 0.025 mL of detection antibody. After 2 hours of incubation at room temperature in dark, 0.025 mL of streptavidin‐PE was added and incubated for another 30 minutes. The mix was then washed twice and analyzed in flow cytometry. The absolute concentrations in the serum were calculated to known standards.

### 4.7 Statistics

We used descriptive statistics (means and standard deviations or median and ranges) to summarize the data. The relationships between overall survival time and the ratio of CD4+CAR‐T/CD8+CAR‐T as well as cytokines (IL‐8, IL‐18, and MCP‐1) and the blast percentage in BM, respectively, were analyzed by linear regression models using the generalized estimating equations method. Pearson correlation was used to test the statistical relationship between two variables. Data were plotted using OriginPro7.5. The survival curve and progression‐free survival (PFS) were determined by the Kaplan‐Meier method. *P* value < .05 was considered to be statistically significant.

## CONFLICT OF INTEREST

J. Li is a founder of Hebei Senlang Bio. Co. Ltd. J.Y.H., Q.W., L.W., Y.L., M.B., and Y.W. are employees of Hebei Senlang Bio. Co. Ltd. The other authors disclosed no potential conflicts of interest.

## AUTHOR CONTRIBUTION

F.M., H.D., F.X., X.W., Q.W., L.W., M.B., J. Luo, and J. Li performed the research. J.H., Y.L., M.B., Y.W., and J. Li analyzed the results. J.H. and J. Li wrote the paper.

## Supporting information

Figure S1:
The flowchart of CAR‐19 manufacture.

Figure S2:
Overall survival and disease free survival of patients receiving CAR‐19.

Figure S3:
The correlation analysis of the blast percentage in bone marrow and the degree of CRS.

Figure S4:
The correlation analysis of the peak concentration of IFN‐γ, IL‐6, IL‐8, and IL‐18 in peripheral blood to the degree of CRS.

Figure S5:
The correlation analysis of the peak concentration of IL‐8, IL‐18, and MCP‐1 in peripheral blood to the blast percentage in peripheral blood.

Figure S6:
The correlation analysis of the peak concentration of IL‐1β, INF‐γ, IL‐6 in peripheral blood to the blast percentage in peripheral blood or bone marrow.

Figure S7:
The kinetics of CAR copy number, percentage of BM blast, and the percentage of CD19 positive cells in BM of patient 9

Table S1: Summary of the adverse effects
Table S2: Characteristic of CAR19
