# Supplementary figures and images for: Evidence of long‐lasting anti‐CD19 activity of engrafted CD19 chimeric antigen receptor–modified T cells in a phase I study targeting pediatrics with acute lymphoblastic leukemia
Source: Hematol Oncol. 2019 Sep 15;37(5):601–8. doi: 10.1002/hon.2672 (PMC6973049; doi:10.1002/hon.2672)

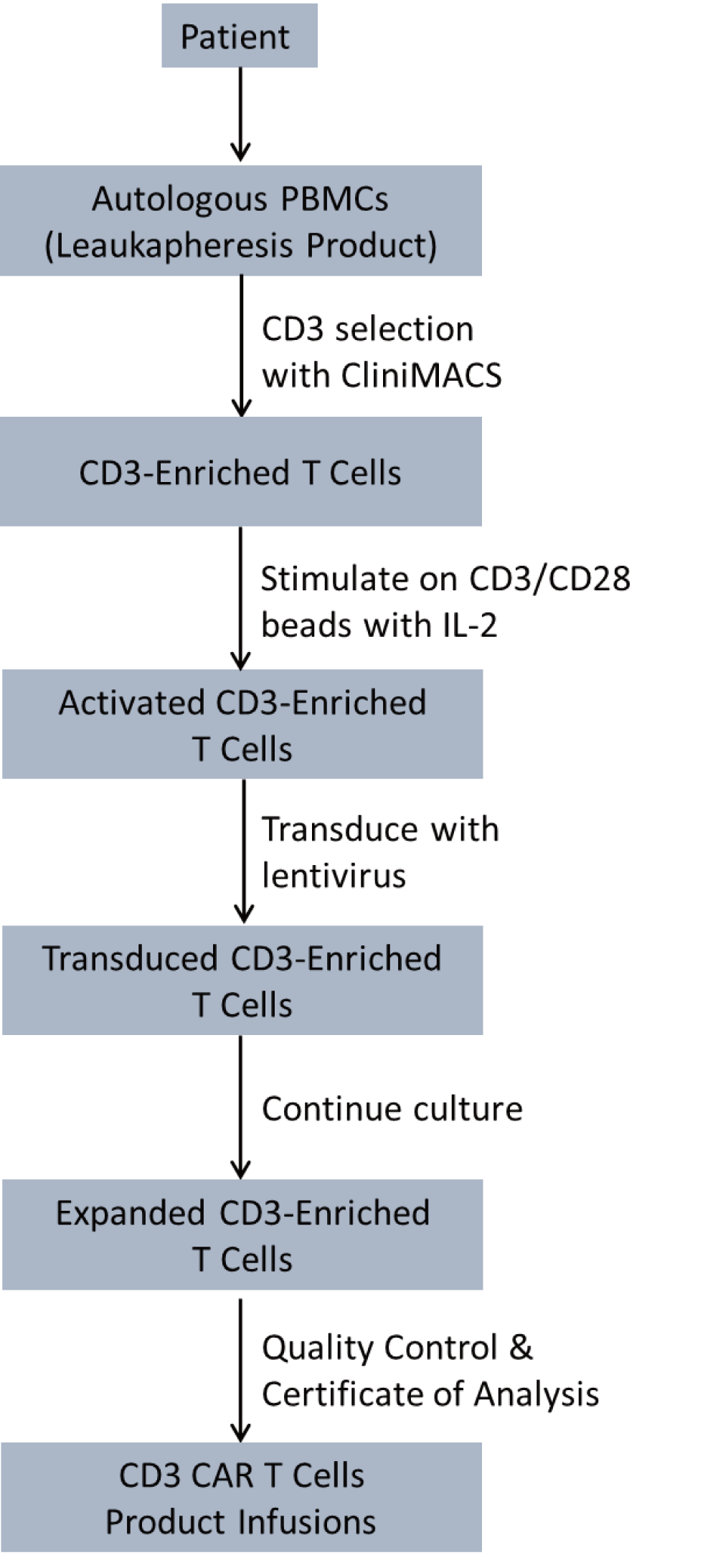

Supplement: Supplementary file 1 — Figure S1: The flowchart of CAR‐19 manufacture. [file HON-37-601-s001.png]

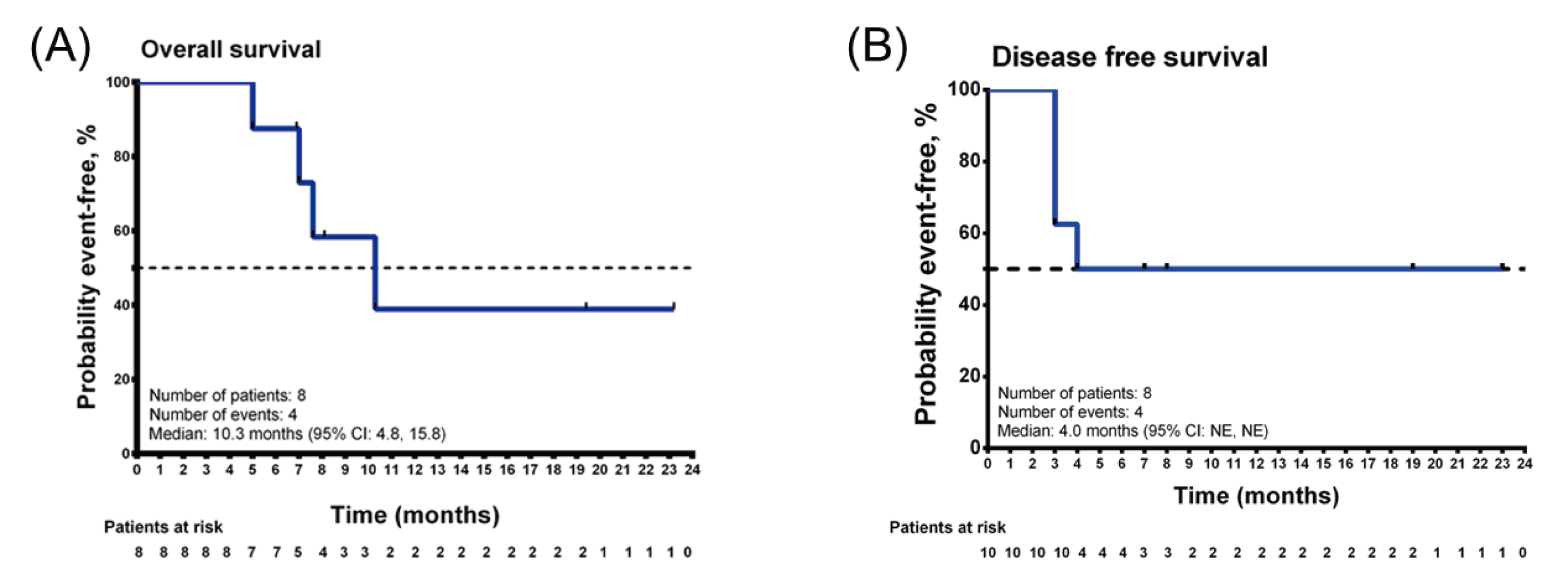

Supplement: Supplementary file 2 — Figure S2: Overall survival and disease free survival of patients receiving CAR‐19. [file HON-37-601-s002.png]

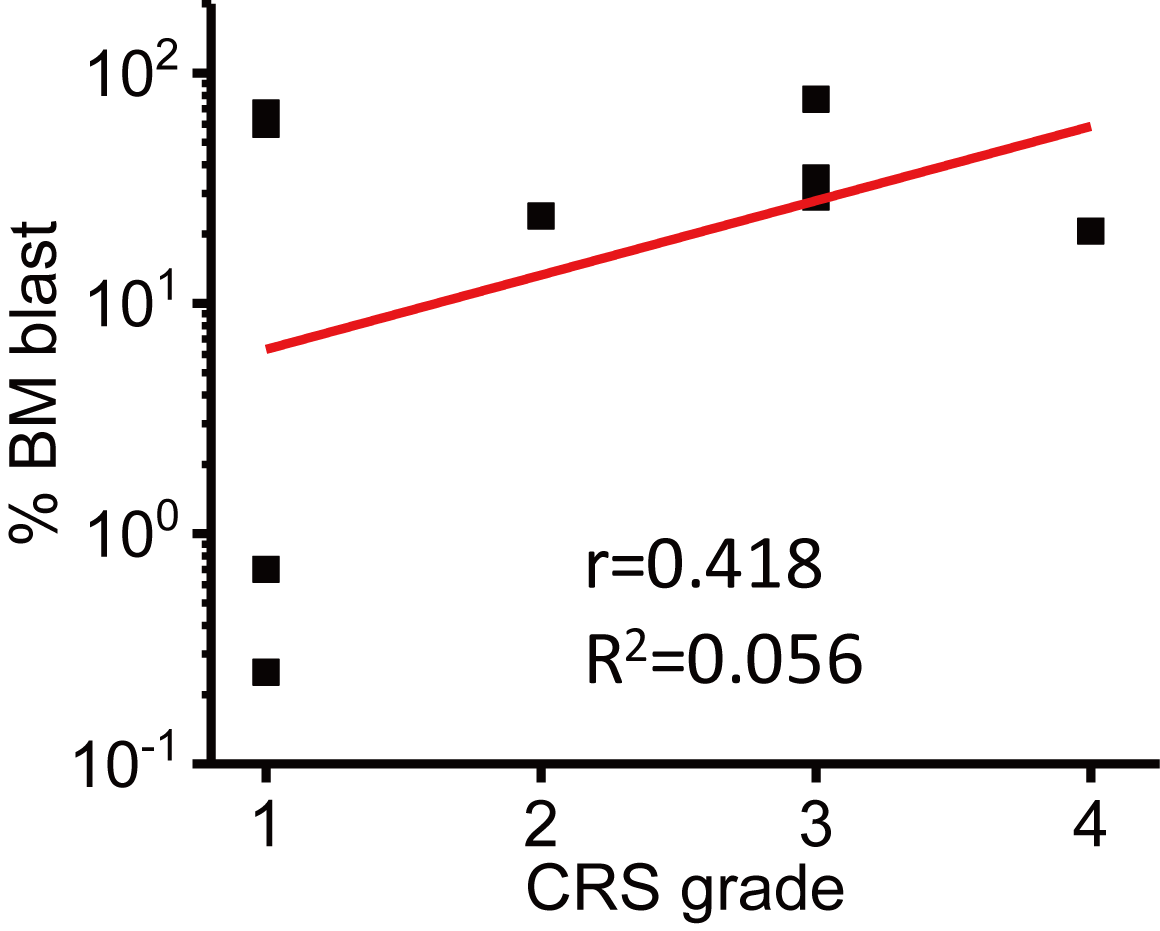

Supplement: Supplementary file 3 — Figure S3: The correlation analysis of the blast percentage in bone marrow and the degree of CRS. [file HON-37-601-s003.png]

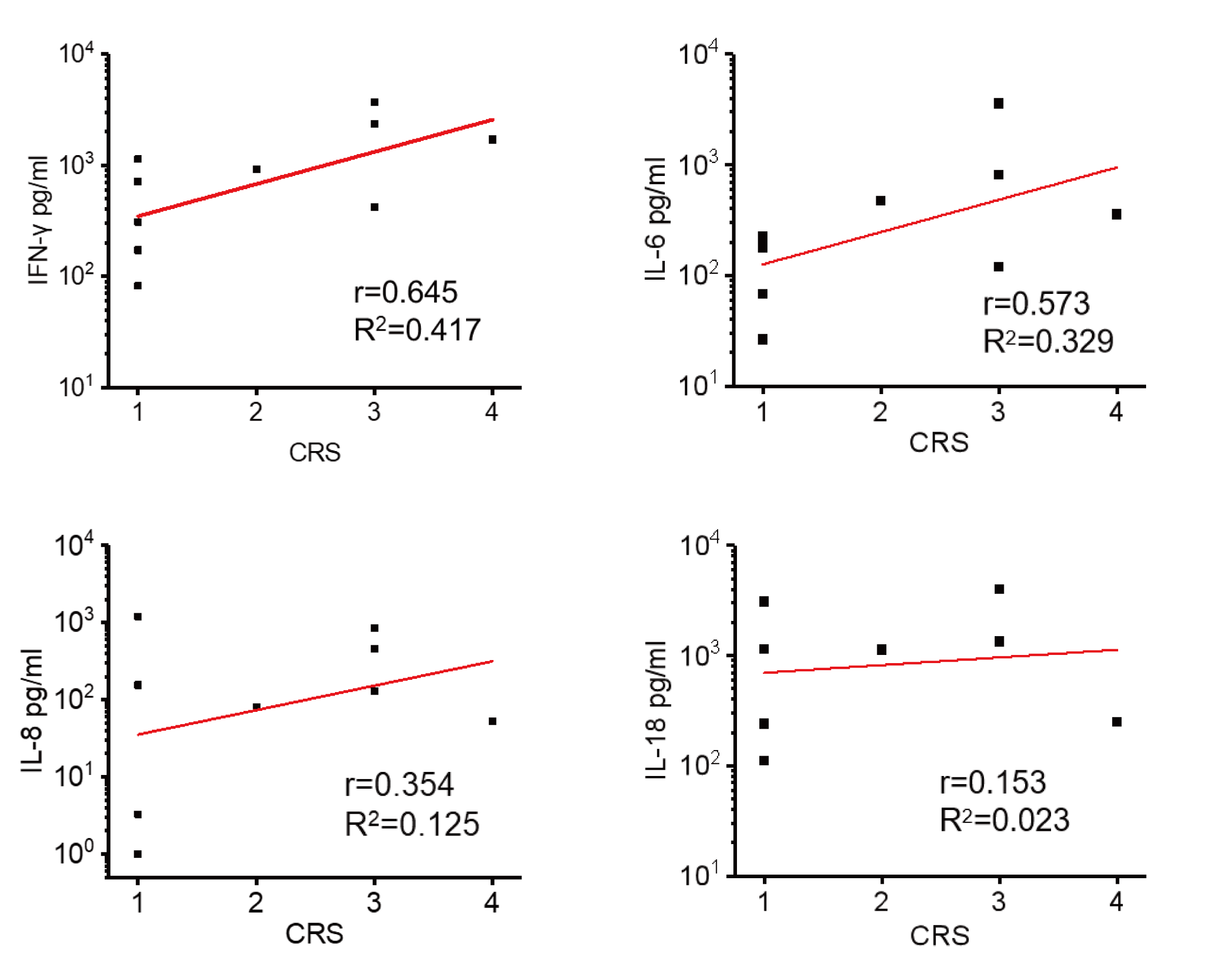

Supplement: Supplementary file 4 — Figure S4: The correlation analysis of the peak concentration of IFN‐γ, IL‐6, IL‐8, and IL‐18 in peripheral blood to the degree of CRS. [file HON-37-601-s004.png]

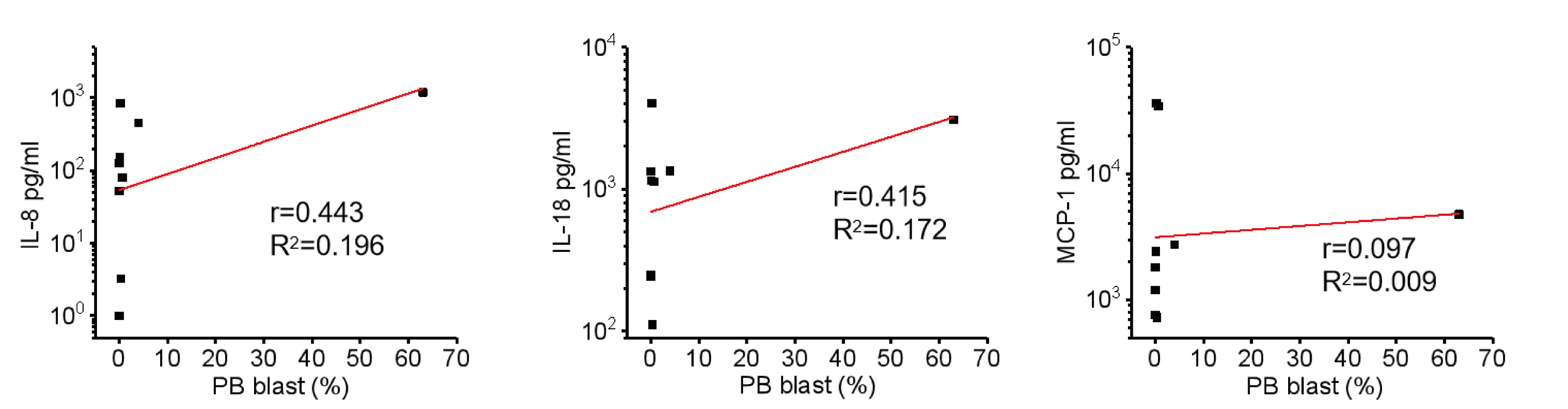

Supplement: Supplementary file 5 — Figure S5: The correlation analysis of the peak concentration of IL‐8, IL‐18, and MCP‐1 in peripheral blood to the blast percentage in peripheral blood. [file HON-37-601-s005.png]

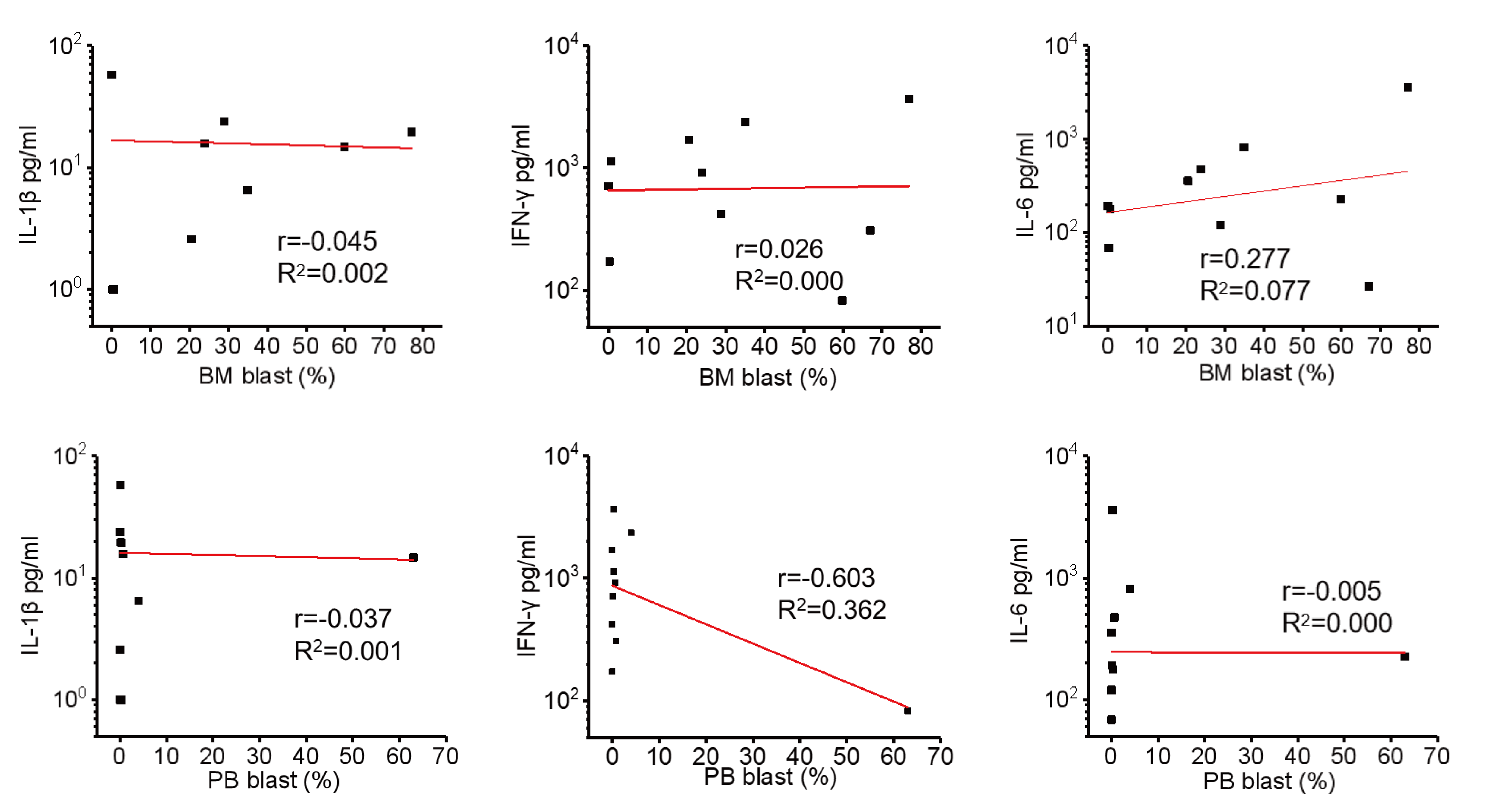

Supplement: Supplementary file 6 — Figure S6: The correlation analysis of the peak concentration of IL‐1β, INF‐γ, IL‐6 in peripheral blood to the blast percentage in peripheral blood or bone marrow. [file HON-37-601-s006.png]

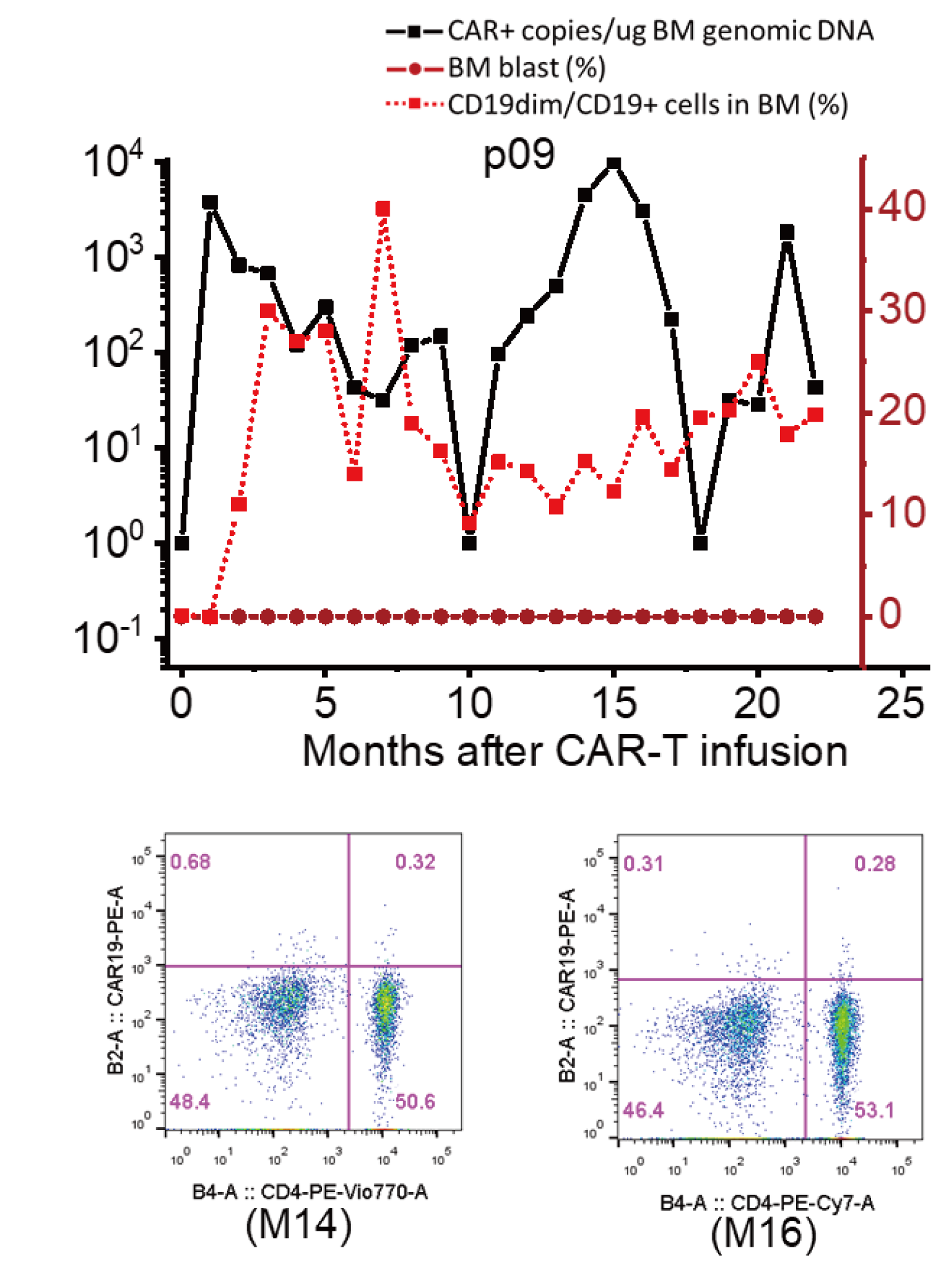

Supplement: Supplementary file 7 — Figure S7: The kinetics of CAR copy number, percentage of BM blast, and the percentage of CD19 positive cells in BM of patient 9 [file HON-37-601-s007.png]
