# Supplementary material for: Evidence of long‐lasting anti‐CD19 activity of engrafted CD19 chimeric antigen receptor–modified T cells in a phase I study targeting pediatrics with acute lymphoblastic leukemia
Source: Hematol Oncol. 2019 Sep 15;37(5):601–8. doi: 10.1002/hon.2672 (PMC6973049; doi:10.1002/hon.2672)
Supplement: Supplementary file 8 — Table S1: Summary of the adverse effects Table S2: Characteristic of CAR19 [file HON-37-601-s008.docx]

| **Supplementary Table 1.** | | | | |
| --- | --- | --- | --- | --- |
| **Event** | Any, n (%) | Grade 3, n (%) | Grade 4, n (%) | Grade 5,n (%) |
| **CRS，any** | 10 (100) | 3 (30) | 0 | 1 (10) |
| **Pyrexia** | 1 (10) | 4 (40) | 0 | 0 |
| **Hypotension** | 3 (30) | 1 (10) | 0 | 0 |
| **tachycardia** | 7 (70) | 0 | 0 | 0 |
| **Acute kidney injury** | 1 (10) | 1 (10) | 0 | 0 |
| **Cardiac failure** | 2 (20) | 1 (10) | 1 (10) | 0 |
| **Headache** | 3 (30) | 0 | 0 | 0 |
| **Hypoxia** | 6 (60) | 4 (40) | 0 | 0 |
| **metabolic acidosis** | 2 (20) | 0 | 2 (20) | 0 |
| **Headache** | 3 (30) | 0 | 0 | 0 |
| **Dizziness** | 1 (10) | 0 | 0 | 0 |
| **Aphasia** | 0 | 0 | 0 | 0 |
| **Ataxia** | 1 (10) | 0 | 0 | 0 |
| **Epilepsy** | 6 (60) | 3 (30) | 0 | 0 |
| **Dyscalculia** | 0 | 0 | 0 | 0 |
| **Myoclonus** | 0 | 0 | 0 | 0 |
| **Insomnia** | 0 | 0 | 0 | 0 |
| **Restlessness** | 0 | 0 | 0 | 0 |
| **Delirium** | 2 (20) | 2 (20) | 0 | 0 |
| **neurotoxicity，any** | 6 (60) | 5 (50) | 1 (10) | 0 |
| **Encephalopathy** | 3 (30) | 1 (10) | 1 (10) | 0 |
| **Tremor** | 0 | 0 | 0 | 0 |
| **somnolence** | 1 (10) | 0 | 0 | 0 |
| **agitation** | 0 | 0 | 0 | 0 |
| **Aphasia** | 0 | 0 | 0 | 0 |
| **Delirium** | 2 (20) | 2 (20) | 0 | 0 |
| **Dizziness** | 1 (10) | 0 | 0 | 0 |
| **hallucination** | 0 | 0 | 0 | 0 |
| **Restlessness** | 0 | 0 | 0 | 0 |

| **Supplementary Table 2. Characteristics of Senl-B19** | | | | | | | | | | |
| --- | --- | --- | --- | --- | --- | --- | --- | --- | --- | --- |
|  | **Patient No.** | | | | | | | | | |
|  | 3 | 4 | 9 | 19 | 21 | 26 | 34 | 44 | 57 | 68 |
| **CAR T cells (10^6^/kg)** | 3.6 | 1.2 | 1.19 | 1.43 | 1.5 | 2.1 | 4.7 | 25 | 3 | 6.7 |
| **CD4 T cells (%)** | 43.75 | 19.8 | 60.92 | 23.3 | 33.82 | 34.9 | 26.69 | 23.5 | 30.4 | 26.5 |
| **CD8 T cells (%)** | 55.45 | 79.3 | 40.42 | 81.1 | 51.62 | 64.8 | 78 | 77 | 68.7 | 71.8 |
| **CD4/CD8 T cell ratio** | 0.78 | 0.25 | 1.51 | 0.29 | 0.65 | 0.54 | 0.34 | 0.31 | 0.44 | 0.37 |
| **CD4/CD8 CAR T cell ratio** | 1.64 | 0.39 | 1.75 | 0.47 | 1.17 | 0.69 | 0.45 | 0.72 | 0.7 | 0.32 |
| **PD1 in CD3 CAR T cells (%)** | N/A | 2.02 | N/A | 1.2 | 0.33 | 0.81 | 0.6 | 0.165 | N/A | 0.24 |
| **Manufacturing time (days)** | 14 | 14 | 14 | 14 | 14 | 14 | 16 | 14 | 15 | 14 |
